# Congenital myasthenic syndrome secondary to pathogenic variants in the *SLC5A7* gene: report of two cases

**DOI:** 10.1186/s12920-024-01977-6

**Published:** 2024-08-12

**Authors:** Javier A Muntadas, Martin R Hyland, Maria Del Rosario Ortolá Martínez, Jaime N Young, Jessica X Chong, Michael J Bamshad, Ricardo A. Maselli

**Affiliations:** 1 Pediatric Neurology, Hospital Italiano, Gascon 450. Capital Federal, Buenos Aires, 4959-0200 Argentina; 2 Department of Genome Sciences, University of Washington, William H. Foege Hall, 3720 15th Ave NE, Seattle, WA 98195 USA; 3 Brotman Baty Institute for Precision Medicine, 1959 NE Pacific St, Seattle, WA 98195 USA; 4 Division of Genetic Medicine, Department of Pediatrics, University of Washington, 4245 Roosevelt Way NE, Seattle, WA 98105 USA; 5 Department of Neurology, University of California Davis, 1515 Newton Court, Davis, CA 95618 USA

**Keywords:** Congenital myasthenic syndromes, Presynaptic, Episodic apnea, Choline transporter 1, *SLC5A7*

## Abstract

**Background:**

Congenital Myasthenic Syndromes (CMS) are rare genetic diseases, which share as a common denominator muscle fatigability due to failure of neuromuscular transmission. A distinctive clinical feature of presynaptic CMS variants caused by defects of the synthesis of acetylcholine is the association with life-threatening episodes of apnea. One of these variants is caused by mutations in the *SLC5A7* gene, which encodes the sodium-dependent HC-3 high-affinity choline transporter 1 (CHT1). To our knowledge there are no published cases of this CMS type in Latin America.

**Case presentation:**

We present two cases of CHT1-CMS. Both patients were males presenting with repeated episodes of apnea, hypotonia, weakness, ptosis, mild ophthalmoparesis, and bulbar deficit. The first case also presented one isolated seizure, while the second case showed global developmental delay. Both cases, exhibited incomplete improvement with treatment with pyridostigmine.

**Conclusions:**

This report emphasizes the broad incidence of CMS with episodic apnea caused by mutations in the *SLC5A7* gene and the frequent association of this condition with serious manifestations of central nervous system involvement.

## Background

Congenital Myasthenic Syndromes (CMS) are a complex group of diseases in which the function of the neuromuscular junction (NMJ) is altered by one or multiple mechanisms, causing muscle weakness and fatigability. Most often, symptoms appear early in life, at birth or even prenatally [1, 2]; however, the clinical manifestations of CMS can start at any time in life. These diseases are caused by defects of genes encoding various proteins that are essential for neuromuscular transmission.

From the first complete description to present, mutations in more than 30 genes have been found to associate with the pathogenesis of CMS [3, 4]. Although CMS are infrequent diseases, they have been reported worldwide [1, 2]. According to the location of the protein encoded by the defective gene, CMS are classified as presynaptic, synaptic, and postsynaptic types with additional variants that involve all three compartments [2]. Postsynaptic forms due to mutations in genes transcribing the adult subunits of the acetylcholine receptor are the most frequent types of CMS [2].

Although all CMS share in varied degree the clinical characteristics described above, those affecting the synthesis of acetylcholine (ACh) have a particular phenotype characterized by recurrent episodes of apnea. These variants correspond to 6–7% of all CMS types [2] and can result from mutations in the genes encoding the choline acetyltransferase *(CHAT)* [5], the vesicular ACh transporter (VAChT) *(SLC18A3)* [6], and the sodium-dependent HC-3 high-affinity choline transporter 1 (CHT1) *(SLC5A7)* [7]. While the CMS caused by mutations in *CHAT* is the most common variant of this subtype of CMS, in recent years, several authors have reported mutations in *SLC5A7* as another frequent cause of CMS with episodic apneas [7–11].

The translation product of *SLC5A7* is the CHT1, which is responsible for choline reuptake in the presynaptic terminal after the cleavage of ACh by the enzyme acetylcholinesterase in the synaptic cleft [10, 11]. There are also dominant mutations of this gene causing type VII hereditary distal motor neuropathy, which has different clinical manifestations than those in patients with CMS with episodic apnea [12]. We describe here the first two cases of CMS with episodic apnea caused by mutations in the *SLC5A7* gene in Latin America and emphasize the frequent association of this genetic disorder with serious manifestations of central nervous system (CNS) involvement.

## Case presentation

This study was approved by the Ethical Committee of the Hospital Italiano. Signed consents to participate and for publication of the case reports and images were obtained from the legal representatives of all the participants.

### Case 1

This male patient, who is currently a 36-month-old, was the first child of a healthy non-consanguineous couple, of Polish and Spanish ancestry. The pregnancy was uncomplicated, but the delivery was performed at term by cesarean section because of the finding of a dilated middle cerebral artery by doppler ultrasound. At birth his Apgar score was normal, he had no joint contractures, and his weight was adequate for gestational age. In the first hour of life, he presented an episode of apnea with generalized cyanosis, which resolved with the administration of supplemental oxygen by nasal cannula. At 6 h, he presented a paroxysmal event characterized by myoclonic jerks of the right hemi-body, interpreted as a possible seizure, which spontaneously resolved. He was transferred to the neonatal intensive care unit (NICU) for continued care. During hospitalization, bilateral ptosis, hypotonia, weakness, laryngeal stridor, dysphonic crying, and weak sucking were observed. An EMG was performed and revealed 13% decrement of compound muscle action potential amplitudes during stimulation of the ulnar nerve at 2 Hz. An EEG and a brain MRI were both normal. After 21 days he was discharged, but readmitted 24 h later, due to an episode of apnea associated with feeding. In the NICU he presented 5 additional episodes requiring the insertion of an orogastric tube. Blood collection for a whole exome sequencing (WES) was completed, and the patient was discharged home.

At 2 months of age, he presented respiratory arrest, for which he was admitted to the pediatric intensive care unit (PICU) and required tracheostomy with prolonged mechanical ventilation.

The WES analysis informed two unreported heterozygous variants in the *SLC5A7* gene (NM_021815.5:c) associated with CMS. One variant was *c.178 + 2T > C* considered pathogenic by the Mutation Taster and Human Splicing Finder software [13, 14]. The other variant *c.1448 C > T*, p.Ala483Val, was considered pathogenic by CADD, MutationAssessor, and MutationTaster, but tolerated by Polyphen2-HVAR, and SIFT (Table 1).

**Table 1:** Genetic studies and clinical features

Genetic and clinical findings	Case 1	Case 2
*SLC5A7* mutations	*c.178 + 2T > C* *c.1448 C > T* p.Ala483Val	*c.1207T > C* p.Tyr403His *c.1349G > A* p.Gly450Glu
gender	male	male
current age	3-year-old	17-year-old
pregnancy	uncomplicated	uncomplicated
arthrogryposis	no	no
onset of symptoms	first day of life	first month of life
ptosis	yes	yes
ophthalmoparesis	yes	yes
hypotonia	yes	yes
weakness	yes	yes
episodes of apnea	yes	yes
seizures	yes	no
developmental delay	no	yes
behavioral changes	no	yes
tracheostomy	yes	yes
response to pyridostigmine	favorable	favorable

**Fig. 1:**
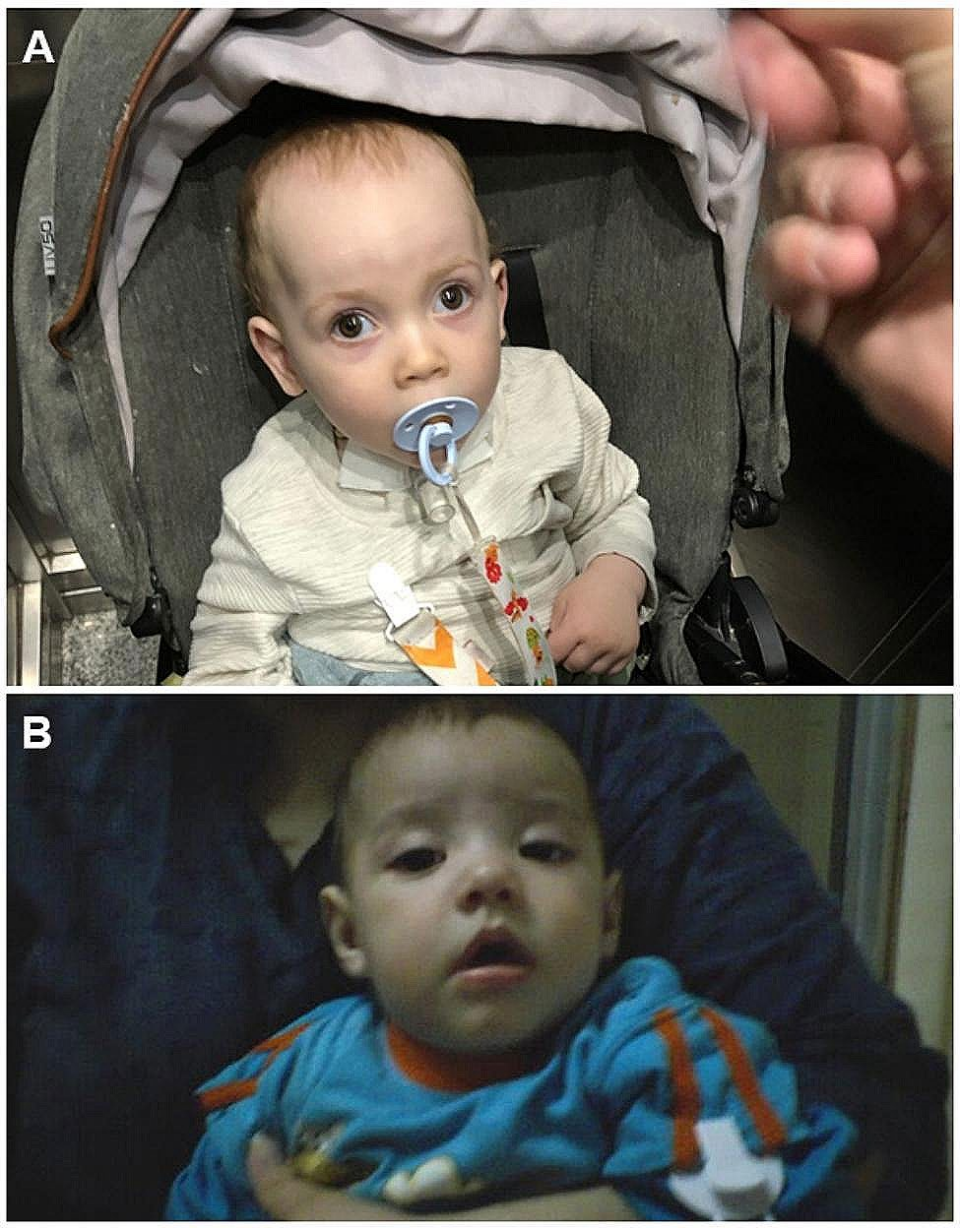
Clinical features. **A**. Patient 1 displaying strabismus and compensatory head tilt. **B** Patient 2 displaying bilateral ptosis

With the tentative diagnosis of CMS, treatment with pyridostigmine 5 mg every 4 h was started, with good clinical response. The patient is now 3-year-old and displays only mild hypotonia and occasional divergent strabismus (Fig. 1A).

### Case 2

**Fig. 2:**
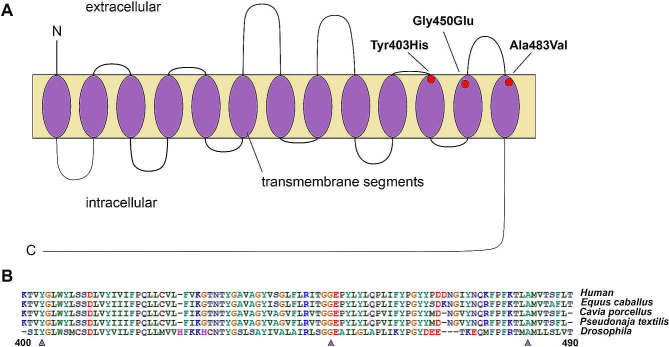
Schematic view of human CHT1 showing the position of the identified mutations. **A**. Variants Ala483Val along with the splice junction mutation *c.178 + 2T > C* (not shown in the drawing) were found in patient 1, while the Tyr403His and Gly450Glu were identified in patient 2. Notice that the three amino acid changes involve transmembrane segments of the CHT1 protein. (modified from reference Haga, 2014). 15**B**. Alignment of the amino acid sequence of the human CHT1 encompassing the identified mutations (arrowheads) with that of orthologous genes from mammals (horse and Guinea pig), vertebrate (easter brown snake) and insect (fruit fly). Notice that the amino acid sequences are well conserved

The second patient, a male born in Paraguay, was the second child of a healthy non-consanguineous parents. The pregnancy was uncomplicated. He was born at 38 weeks of gestational age, without joint contractures and with a vigorous Apgar score. At 48 h he was discharged from the hospital. A few days following birth, bilateral ptosis, ophthalmoparesis, hypotonia, generalized weakness and significant head lag on traction were observed. He also presented several episodes of apnea throughout his first month of life. This was interpreted as recurrent laryngitis, secondary to gastroesophageal reflux and he underwent Nissen fundoplication surgery. At 3 months, he suffered a respiratory arrest, for which he was admitted to the PICU and required tracheostomy with mechanical ventilation. A muscle biopsy, and a single-fiber electromyography (SFEMG) study were performed in the first year of life. The muscle biopsy showed no diagnostic features, but the SFEMG study showed signs of failure neuromuscular transmission. He has global developmental delay, but he is currently able to communicate using a limited vocabulary. He is able to count single digits and to recognize geometrical figures. However, he is unable to read or write and requires assistance with activities of daily living. Genetic studies were performed, using a comparative genomic hybridization (CGH) microarray, and WES. The CGH demonstrated a microdeletion in chromosome 6q22.32 (Chr6:126241510–126835279), also present in her healthy mother, therefore not considered to be pathogenic. By contrast, the WES demonstrated two unreported pathogenic variants in *SLC5A7* (NM_021815.5:c): *c.1207T > C*, p.Tyr403His, and *c.1349G > A*, p.Gly450Glu (Table 1; Fig. 2), each one shared with each one of his parents. Both variants were considered likely pathogenic based on CADD scores (CADD 24.7; CADD 32.0).

Treatment was started with pyridostigmine, with a good but incomplete clinical response. Thus, ephedrine was added but subsequently discontinued due to restlessness. Choline supplement, and donepezil were later added with a clinical improvement of cognitive function. The patient evolved favorably from the respiratory standpoint, but currently presents a global developmental delay, with serious behavioral derangement. Occasional bilateral ptosis has also been observed since initiation of treatment (Fig. 1B).

## Discussion and conclusions

We present two cases of presynaptic CMS, caused by pathogenic variants of the *SLC5A7* gene. To date, fewer than 15 cases have been identified worldwide, and none of which have been reported in Latin America [11]. The clinical presentation of our patients was characterized by episodes of apnea in the neonatal period, muscle weakness and generalized hypotonia, ptosis, ophthalmoparesis and bulbar symptoms. The first case also presented an episode of focal seizure in the first hours of life, while the second manifested global developmental delay with notable hyperactivity and behavioral disturbance. In both, the etiological diagnosis was achieved through WES, and they exhibited a good, but partial clinical response to treatment with pyridostigmine.

Congenital Myasthenic Syndromes are a rare entity, with an estimated prevalence of 9 to 10 per million [9]. From the pathophysiological point of view, the defect is found in the postsynaptic level in 75% of cases. The rest of the cases correspond 15% to synaptic forms and 6–8% to presynaptic variants [9]. Among the later, CHT1 deficiency due to *SLC5A7* gene mutations are in second place in prevalence of presynaptic CMS after ChAT deficiency, which represents about 5% of CMS [16].

At the nerve terminal, CHT1 is responsible for choline reuptake, which is the rate-limiting reaction in the synthesis of ACh [17, 18]. In recent years, a series of pathogenic variants in genes linked to CMS have been reported in pediatric patients with neuromuscular disorders as a primary symptom but also with clinical manifestations of CNS involvement, which expands the definition of what was previously interpreted as CMS [19, 20].

Cholinergic neurotransmission is widely present throughout the entire nervous system, so it is expected that the clinical spectrum of this entity be broad. According to the literature, CHT1 deficiency is characterized by onset in the neonatal period, with hypotonia and generalized weakness, ocular symptoms, bulbar compromise, and episodes of apnea. A large proportion of patients also present epilepsy and different types of cognitive developmental delay. For instance, cognitive deficit was described in 3 out of the 5 patients reported by Bauche et al. [7], and in all of the 4 patients reported by Wang et al. [8]. By contrast seizures were reported in only one of the patients described by Wang et al. [8] and in 2 out 5 patients presented by Rodriguez Cruz et al. [9]. These clinical manifestations may result from hypoxic brain damage caused by repeated apneic episodes or may represent a direct consequence of impaired cholinergic transmission in the CNS. Indeed, since CHT1 is also required for the reuptake of ACh in central neurons that participate in executive functions and behavioral control [18], it is not surprising that deficiency of CHT1 has far-reaching consequences than impaired neuromuscular transmission.

Episodes of apnea are always serious because they place the lives of patients at risk. The cases reported to date illustrate the morbidity associated with these events, since most patients require invasive ventilatory support and tracheostomy to maintain effective airway control [7, 9, 20].

The diagnostic suspicion of CMS is mainly based on the presence of relevant symptoms and a positive family history. Nevertheless, since all cases of CMS due to *SCL5A7* mutations thus far described have been recessively transmitted, there is usually no family history of CMS in these patients. For the same reason, affected children carry two pathogenic variants in *SLC5A7*, while the parents, who carry only one pathogenic variant, are asymptomatic and do not require electrodiagnostic testing. Electrophysiology studies demonstrating impaired neuromuscular transmission and blood work showing absence of autoantibodies against proteins of the NMJ are useful. However, the key to the diagnosis lies in the finding of pathogenic variants in genes involved in the structure and function of the NMJ, which is usually achieved by performing a WES [1, 2]. The genetic diagnosis is also important to rule out other forms of CMS, such as those caused by mutation in genes encoding ChAT, Rapsyn, Agrin and ColQ, which can also result in episodes of apnea.

In the CHT1-CMS acetylcholinesterase inhibitors, such as pyridostigmine, by virtue of increasing the amount of ACh available to interact with the receptor, improve neuromuscular transmission [9, 11], However, in some CMS variants such those resulting from mutations in Agrin and ColQ, pyridostigmine can result in an untoward effect. Moreover, since in CHT1-CM the therapeutic response to pyridostigmine is incomplete, sympathomimetic medications, such as Salbutamol and Ephedrine are often needed to support neuromuscular transmission. For this reason, early genetic diagnosis is essential since therapeutic intervention could improve the morbidity and mortality of the disease. In the case of our patients, after administration of pyridostigmine at adequate doses, improvement was observed in both muscular and respiratory function with resolution of apnea episodes. However, in patient 2, there were no observable changes of the behavior or cognitive functions. Similar findings have been described by other authors, although some have described a partial improvement the behavioral dysfunction [7–9, 11]. To explain this phenomenon, it was proposed that the response variability may be due to the severity of the clinical phenotype and the residual CHT1 function, although the latter is not routinely performed in daily clinical practice [10].

## Data Availability

The DNA sequence data analyzed in this study are available in the ClinVar public archive : https://www.ncbi.nlm.nih.gov/clinvar/: [NM_021815.5(SLC5A7):c.178 +2T>C Variation ID: 2687476 Accession: VCV002687476.1, NM_021815.5(SLC5A7):c.1207T>C (p.Tyr403His) Variation ID: 2687766 Accession: VCV002687766.1, NM_021815.5(SLC5A7):c.1349G>A (p.Gly450Glu) Variation ID: 2687767 Accession: VCV002687767.1, NM_021815.5(SLC5A7):c.1448C>T (p.Ala483Val) Variation ID: 2687475 Accession: VCV002687475.1]

## Abbreviations

CMS: congenital myasthenic syndromes
NMJ: neuromuscular junction
ACh: acetylcholine
SLC5A7: solute carrier 5A7 gene
CHAT: choline acetyltransferase gene
VAChT: vesicular acetylcholine transporter
CHT1: choline transporter 1
WES: whole-exome sequencing

## References

[1] Estephan EP, Zambon AA, Thompson R, Polavarapu K, Jomaa D, Töpf A, et al. Congenital myasthenic syndrome: correlation between clinical features and molecular diagnosis. Eur J Neurol. 2022;29(3):833–42. 34749429 10.1111/ene.15173

[2] Engel AG, Shen XM, Selcen D, Sine SM. Congenital myasthenic syndromes: pathogenesis, diagnosis, and treatment. Lancet Neurol. 2015;14(4):420–34. 25792100 10.1016/S1474-4422(14)70201-7 PMC4520251

[3] Engel AG, Lambert EH, Gomez MR. A new myasthenic syndrome with end-plate acetylcholinesterase deficiency, small nerve terminals, and reduced acetylcholine release. Ann Neurol. 1977;1(4):315 – 30. 10.1002/ana.410010403. PMID: 214017. 10.1002/ana.410010403 214017

[4] Ohno K, Ohkawara B, Shen XM, Selcen D, Engel AG. Clinical and Pathologic Features of Congenital Myasthenic Syndromes Caused by 35 Genes-A Comprehensive Review. Int J Mol Sci. 2023;24(4):3730. 10.3390/ijms24043730. PMID: 36835142. 10.3390/ijms24043730 PMC9961056 36835142

[5] Ohno K, Tsujino A, Brengman JM, Harper CM, Bajzer Z, Udd B et al. Choline acetyltransferase mutations cause myasthenic syndrome associated with episodic apnea in humans. Proc Natl Acad Sci U S A. 2001;98(4):2017–2022; 10.1073/pnas.98.4.2017. PMID: 11172068. 10.1073/pnas.98.4.2017 PMC29374 11172068

[6] O’Grady GL, Verschuuren C, Yuen M, Webster R, Menezes M, Fock JM, et al. Variants in SLC18A3, vesicular acetylcholine transporter, cause congenital myasthenic syndrome. Neurology. 2016;87(14):1442–8. PMID: 27590285. 27590285 10.1212/WNL.0000000000003179 PMC5075972

[7] Bauché S, O’Regan S, Azuma Y, Laffargue F, McMacken G, Sternberg D et al. Impaired presynaptic high-affinity choline transporter causes a congenital myasthenic syndrome with episodic apnea. Am J Hum Genet. 2016;99(3):753–761; 10.1016/j.ajhg.2016.06.033. Epub 2016. PMCID: PMC5011057. 10.1016/j.ajhg.2016.06.033 PMC5011057 27569547

[8] Wang H, Salter CG, Refai O, Hardy H, Barwick KES, Akpulat U, et al. Choline transporter mutations in severe congenital myasthenic syndrome disrupt transporter localization. Brain. 2017;140(11):2838–50. 29088354 10.1093/brain/awx249 PMC5844214

[9] Pardal-Fernández JM, Carrascosa-Romero MC, Álvarez S, Medina-Monzón MC, Caamaño MB, de Cabo C. A new severe mutation in the SLC5A7 gene related to congenital myasthenic syndrome type 20. Neuromuscul Disord. 2018;28(10):881–4. 30172469 10.1016/j.nmd.2018.06.020

[10] Rodríguez Cruz PM, Hughes I, Manzur A, Munot P, Ramdas S, Wright R, et al. Presynaptic congenital myasthenic syndrome due to three novel mutations in SLC5A7 encoding the sodium-dependant high-affinity choline transporter. Neuromuscul Disord. 2021;31(1):21–8. 33250374 10.1016/j.nmd.2020.10.006

[11] Rizvi M, Truong TK, Zhou J, Batta M, Moran ES, Pappas J, et al. Biochemical characterization of two novel mutations in the human high-affinity choline transporter 1 identified in a patient with congenital myasthenic syndrome. Hum Mol Genet. 2023. 10.1093/hmg/ddac309. 36611016 10.1093/hmg/ddac309

[12] Barwick KE, Wright J, Al-Turki S, McEntagart MM, Nair A, Chioza B et al. Defective presynaptic choline transport underlies hereditary motor neuropathy. Am J Hum Genet. 2012;91(6):1103–7. 10.1016/j.ajhg.2012.09.019. Epub 2012. PMID: 23141292. 10.1016/j.ajhg.2012.09.019 PMC3516609 23141292

[13] Schwarz JM, Rodelsperger C, Schuelke M, Seelow D. Mutation Taster evaluates disease-causing potential of sequence alterations. Nat Methods. 2010;7(8):575–6. 10.1038/nmeth0810-575. PMID: 20676075. 10.1038/nmeth0810-575 20676075

[14] Desmet FO, Hamroun D, Lalande M, Collod-Béroud G, Claustres M, Béroud C. Human Splicing Finder: an online bioinformatics tool to predict splicing signals. Nucleic Acids Res. 2009;37(9):e67. 10.1093/nar/gkp215. Epub 2009. PMID: 19339519. 10.1093/nar/gkp215 PMC2685110 19339519

[15] Haga T. Molecular properties of the high-affinity choline transporter CHT1. J Biochem. 2014;156(4):181–94. 10.1093/jb/mvu047. Epub 2014 Jul 29. PMID: 25073461. 10.1093/jb/mvu047 25073461

[16] Zhang Y, Cheng X, Luo C, Lei M, Mao F, Shi Z, et al. Congenital myasthenic syndrome caused by a Novel Hemizygous CHAT Mutation. Front Pediatr. 2020;8:185. 10.3389/fped.2020.00185. eCollection 2020.PMID: 32411636. 32411636 10.3389/fped.2020.00185 PMC7198756

[17] Guidry G, Willison BD, Blakely RD, Landis SC, Habecker BA. Developmental expression of the high affinity choline transporter in cholinergic sympathetic neurons. Auton Neurosci. 2005;123(1–2):54–61. 10.1016/j.autneu.2005.10.001. Epub 2005 Nov 8. PMID: 16278103. 16278103 10.1016/j.autneu.2005.10.001 PMC1407245

[18] Cuddy LK, Seah C, Pasternak SH, Rylett RJ. Differential regulation of the high-affinity choline transporter by wild-type and Swedish mutant amyloid precursor protein. J Neurochem. 2015;134(4):769–82. 10.1111/jnc.13167. Epub 2015 Jun 3. PMID: 25970623. 25970623 10.1111/jnc.13167

[19] Ramdas S, Beeson D. Congenital myasthenic syndromes: where do we go from here? Neuromuscul Disord. 2021;31(10):943–54. 34736634 10.1016/j.nmd.2021.07.400

[20] McMacken G, Whittaker RG, Evangelista T, Abicht A, Dusl M, Lochmüller H. Congenital myasthenic syndrome with episodic apnoea: clinical, neurophysiological and genetic features in the long-term follow-up of 19 patients. J Neurol. 2018;265(1):194–203. 29189923 10.1007/s00415-017-8689-3 PMC5760613

